# A Pathognomonic Symptom in the Diagnosis of Tubercular Meningitis

**Published:** 1887-01

**Authors:** J. D. Skeer

**Affiliations:** 681 Washington Boulevard


### A PATHOGNOMONIC SYMPTOM IN THE DIAGNOSIS OF TUBERCULAR MENINGITIS.—*By* J. D. SKEER, M.D.

[Read before the Chicago Pathological Society July 26th, 1886, and referred to a committee, whose report is appended.]

In presenting this short paper on the differential dignosis of tubercular meningitis I will not dwell on the pathology or treatment of the disease, and only briefly on its symptomatology.

The main object of the paper is to call the attention of the society to a symptom which I have never seen mentioned in the literature of the subject, and which, according to my observation, is always present during the first or second stage of tuberculous inflammation of the membranes of the brain. We all know the importance and difficulty in distinguishing between simple and tubercular meningitis in the acute stage, how each progresses from day to day with little variation, having remissions and exacerbations in common. The tuberculous cachexia may not be well marked, and a guarded prognosis is given in the hope that the disease may prove to be of simple inflammatory origin. A few cases do occur in which a diagnosis can be made with reasonable certainty, but with the majority this is impossible.

The symptom to which I allude is a small circle which forms in the iris, near to and completely surrounding the pupillary margin. When it begins to appear, it is very indistinct and resembles a wreath of thin, white clouds, the edge of which extends at first to the free border of the iris. In from twelve to thirty-six hours the whole margin of the iris will be involved, having become of a whitish or yellowish-brown color, and appearing irregular, thickened and somewhat granular. These changes, which commence in both eyes simultaneously, are more apparent in irides of a brown color. In this connection we should remember that some children, especially those born of scrofulous or tuberculous parents, have a light colored circle *on* the free border of the iris, composed of cloudy and white fibres which radiate from the centre toward the circumference. This cloud-like appearance is in some cases very evanescent, hence the necessity of examining the iris at every visit. They may appear in the evening, and by the following morning have given place to the yellow-brown rings caused by the subsequent changes in the tissues. These later rings are permanent and grow narrower and larger with the progressive dilatation of the pupils as the disease advances.

In cases where tubercle bears an ætiological relation to the meningeal trouble, the circles appear early, before or during the decline of the fever which characterizes the first stage of the disease. In cases where the original simple inflammation has, by its nutritive changes, called into activity a latent tubercular tendency, they appear in the second stage of the disease. In no instance have I observed them in a simple acute case.

I first noticed this peculiar symptom some twenty years ago in a case occurring in my own family. At that time I attached no importance to it, but since then I have met with it in twenty or more patients, all of whom died. In two cases an opportunity of making a *post-mortem* examination was afforded, tubercles being found in each instance.

I have never examined the iris under the microscope, but it seems to me to be in harmony with the pathological anatomy of tubercular meningitis to regard this morbid process as an infiltration of tuberculous matter into the perivascular sheaths of the circulus minor, followed by some degenerative change of the tissues of the iris. Nothing, however, but a careful microscopical examination can determine its pathological anatomy.

When the wreaths of white clouds appear in the iris in a case of cerebral meningitis, I consider the question of diagnosis settled beyond all doubt. They are, in my opinion, pathognomonic of tubercular meningitis, and enable us to make a positive diagnosis, and at the same time an unfavorable prognosis.

681 Washington Boulevard.

---

REPORT OF THE COMMITTEE ON DR. SKEER'S PAPER.

Your committee have carefully examined the literature of this subject, and, so far as their examination has extended, have found no mention of the exact condition described by Dr. Skeer. It is true that several authors give lengthy descriptions, among the best of which is Steiner's, of the relation of tubercular meningitis to certain eye symptoms, particularly the deposit of tubercles in the choroid, but the peculiar symptom here described is not mentioned. A deposit of tubercle in the iris is very rare even in the course of acute tuberculosis, and as a primary local condition it is doubtful if it ever occurs. Again, this condition differs so markedly in appearance (granulom of Steiner) from the symptom under consideration, that it is impossible that they should be confounded.

In view of the silence of the authorities on this subject, your committee entered into correspondence with a number of physicians especially interested in ophthalmology and pædiatrics.

Dr. Adolf Alt, of St. Louis, writes: "I have never observed the symptom you refer to, nor has it, so far as I know, ever been described. It would indeed be of great diagnostic value if it could be established as co-existing always and only with tubercular meningitis."

Dr. Marcus P. Hatfield, of Chicago, writes: "I have no personal knowledge of any such symptom as you describe in tubercular meningitis. Neither do I know of any allusion to it in the literature of pædiatrics. The only reference, that I have met with, to any such condition may be found in Steiner, who says, after speaking of injection of the conjunctivæ in these cases, 'Keratomalacia in one or both eyes sometimes occurs.' Is it possible that this is what Dr. Skeer has observed?"

Dr. H. Knapp, of New York City, writes: "Your favor concerning Dr. Skeer's communication of a new symptom of tubercular meningitis has interested me greatly. I am not aware of having seen anything of the kind referring to tuberculosis, nor have I heard of it. It is difficult, however, to judge of such conditions without seeing them in nature. I shall anxiously look forward to the appearance of the full report."

Dr. Bock, of Vienna, Austria, writes: "I do not remember to have seen or read of a case like the one described. You know how difficult a pathological question is when one has not seen the particular case. I believe the appearance you mention is only to be explained on the theory of a marked congestion of the tissue of the sphincter pupillæ and its neighborhood, with singular (auffallend) and rapid exudation of the coloring matter of the blood into the tissues. This is an explanation of the color changes, and is, in my opinion, what most frequently happens. But one must exercise great reserve in accepting any explanation of this kind. No special eye trouble associated with cerebro-spinal meningitis has, so far as I know, been definitely announced."

Your committee have had an opportunity of seeing one case,* in the service of Dr. I. N. Danforth, at St. Luke's hospital.

This was a case of tabes mesenterica, with secondary involvement of the meninges of the brain. The eye appearances were declared by Dr. Skeer to be quite characteristic, though somewhat irregular in the fact that they existed in the prodromic stage. The light colored circles were symmetrical and distinct, but doubt is thrown on this observation, as they were not seen to *appear*. In other words, it is the development of this condition in the iris which will enable us to distinguish between the appearance of the eye in some scrofulous and cachectic children, and that in tubercular meningitis.

Whatever explanation of the phenomenon may be adopted, we hardly feel justified, from present evidence, in assuming that this is a distinct and peculiar symptom, only present in tubercular meningitis. If we assume that the appearances are due to a perivascular inflammation of tubercular origin, the importance of it as a symptom rests upon the known inconstant relations of other observed eye symptoms to tubercular inflammation of the brain. Tubercles of the choroid often exist without involvement of the meninges, and, again, tubercular meningitis may go on to a fatal termination without any observable invasion of the eye.

The suggestion of a plastic exudation by Bock furnishes a possible explanation. It will be observed that of the twenty fatal cases reported, in only two was a post mortem examination had. It is assuming too much to infer that, because the

* Since the preparation of this report a case of undoubted tubercular meningitis has been under the care of one of the members of the committee. The peculiar symptom under consideration did not appear in this case, though it was carefully looked for during the whole course of the disease, which terminated fatally.

remaining eighteen died, the disease was with them necessarily of tubercular origin. The intimate relation between the circulation of the brain and eye might lead to this inflammatory exudate in the iris *when the inflammation within the cranium had become so severe as to involve the functional integrity of the brain.* Additional support is given to this view by the fact that all the cases, in which this symptom was *not* observed, recovered. While it may not enable us to diagnosticate the character of the inflammation, it may be of great importance in indicating its severity.

Whatever may ultimately prove to be the value of the symptom, we are justified in regarding it as a strictly original observation, which may be termed "Skeer's symptom," until name is found which expresses its pathological relations.

HENRY M. LYMAN,
I. N. DANFORTH,
E. I. HOLMES,
HAROLD N. MOYER.

